# Working toward Personalized Intervention Advice: A Survey Study on Preference Heterogeneity in Patients with Breast Cancer–Related Fatigue

**DOI:** 10.1177/23814683241309676

**Published:** 2025-01-13

**Authors:** Lian Beenhakker, Kim A. E. Wijlens, Christina Bode, Miriam M. R. Vollenbroek-Hutten, Sabine Siesling, Janine A. van Til, Annemieke Witteveen

**Affiliations:** Department of Biomedical Signals and Systems, Technical Medical Centre, University of Twente, Enschede, The Netherlands; Department of Biomedical Signals and Systems, Technical Medical Centre, University of Twente, Enschede, The Netherlands; Department of Psychology, Health and Technology, University of Twente, Enschede, The Netherlands; Department of Biomedical Signals and Systems, Technical Medical Centre, University of Twente, Enschede, The Netherlands; Board of Directors, Medisch Spectrum Twente, Enschede, The Netherlands; Department of Health Technology and Services Research, Technical Medical Centre, University of Twente, Enschede, The Netherlands; Department of Research and Development, Netherlands Comprehensive Cancer Organisation (IKNL), Utrecht, The Netherlands; Department of Health Technology and Services Research, Technical Medical Centre, University of Twente, Enschede, The Netherlands; Department of Biomedical Signals and Systems, Technical Medical Centre, University of Twente, Enschede, The Netherlands

**Keywords:** breast cancer, patient preferences, cancer-related fatigue, best-worst scaling

## Abstract

**Introduction.** Many breast cancer survivors experience cancer-related fatigue (CRF), and several interventions to treat CRF are available. One way to tailor intervention advice is based on patient preferences. In this study, we explore preference heterogeneity regarding between-attribute and within-attribute preferences. In addition, we propose simple decision rules to match preferences to interventions. **Methods.** Nine attributes were included with dichotomized levels. Participants selected their preferred level per attribute and ranked the attributes using best-worst scaling. Between-attribute and within-attribute preferences were determined, together with their heterogeneity. Using decision rules, matching scores were calculated for a hypothetical intervention. **Results.** Sixty-seven breast cancer survivors completed the survey. They were on average 52 y old, 4.5 y after diagnosis, experienced CRF (6.5–7.2/10) on 3 dimensions (physical, mental, and emotional), and 43% already followed an intervention for CRF. Overall, participants ranked *costs* highest. Next to *costs*, *proveneffectiveness* and *type of intervention* were also frequently ranked first. Only 13 participants (19%) shared the most common preference pattern of shorter interventions, daily sessions, shorter session time, a psychosocial intervention, no anonymity, and contact with a therapist and peers. Matching scores for a hypothetical intervention with attributes corresponding with the overall within-attribute preferences varied from 44% to 100%. **Conclusion.** A large heterogeneity in preferences of breast cancer survivors for CRF intervention attributes was demonstrated. Using simple decision rules, the effect of this heterogeneity on linking preferences to interventions with matching scores was demonstrated. **Implications.** Personalization of intervention advice is necessary due to preference heterogeneity. Tailored advice can result in higher involvement of patients in decision making, intervention adherence and satisfaction, and subsequently a potential higher quality of life after breast cancer.

**Highlights:**

- Many breast cancer survivors experience cancer-related fatigue for which many interventions exist.
- Our results show large preference heterogeneity in breast cancer patients’ preferences for attributes of eHealth interventions.
- Based on this preference heterogeneity, intervention advice for cancer-related fatigue after breast cancer can be personalized, ultimately improving quality of life after breast cancer.

One of the most reported long-term effects after breast cancer diagnosis and treatment is cancer-related fatigue (CRF). Up to 40% of the breast cancer survivors still experience high levels of fatigue 5 y after diagnosis.^1,2^ Fortunately, there are many interventions that can treat these survivors with their struggles. The overall effectiveness of these CRF interventions are described in many (umbrella) reviews.^3–7^ Still, there is not one gold standard intervention that works best for everyone.^8^ It is expected that if a patient follows an intervention that fits their preferences, the satisfaction with, uptake of, and adherence to the intervention increases, whereas dropout rates decrease.^9–12^

Therefore, in current studies, patient preferences are considered when developing a new intervention. Important attributes or attribute levels for new interventions follow from preference studies such as a discrete-choice experiment^13^ or a best-worst scaling study.^14^ Preferences vary between individuals and, this preference heterogeneity was found in various studies. Patient characteristics such as time since diagnosis, age, gender, and stage of cancer influence preferences for intervention modality, how and where information is provided, and whether caregivers and/or peers are involved.^14–16^ When an intervention is developed based on preference study, it is not always reported how variation in preferences was incorporated when developing an intervention.^14,17^

However, preference heterogeneity exists on more than attribute, and instead of developing new interventions, patient preferences can be used to advise patients an already existing intervention. In the Personalized cAnceR TreatmeNt and caRe (PARTNR) project, we aim to support breast cancer patients and survivors with CRF by providing them with a personalized intervention advice. One of the aspects to personalize upon is thus patient preferences. We have already shown that eHealth interventions vary on attributes, such as duration, intensity, and contact with health care professionals.^18^ If breast cancer survivors have varying preferences for these specific attributes, this will allow us to develop decision rules and personalize upon these preferences.

There are different quantitative methods to elicit patient preferences.^19^ These are 1) discrete-choice-based methods, including discrete choice experiments (DCE)^13,20^; 2) ranking methods, including best-worst scaling (BWS) case 1 and 2^14,21^; 3) indifference methods, for example, standard gamble^22,23^; and 4) rating methods, for example, analytic hierarchy process (AHP).^20,24^ Using these methods, it can be determined what patients prefer related to the relative importance of attributes (between-attribute preferences) and the outcomes on attribute level (within-attribute preferences). To determine individual preferences, BWS is better suited than DCE.^25^ AHP can also be used to elicit individual preferences^26^ but might lead to many comparisons with increasing number of attributes and their levels.

Breast cancer patients’ preferences for attributes of eHealth interventions are currently unknown. Therefore, we cannot yet develop and validate decision rules to personalize intervention advice for breast CRF. We hypothesize that there will be preference heterogeneity in preferences for attributes of eHealth interventions for breast CRF. However, the extent of this heterogeneity is unclear. The variation in preferences will allow us to link this variation in preferences to the variations in attributes of eHealth interventions. The aim of this study is thus to explore preference heterogeneity regarding 1) the relative importance of the attributes and 2) the outcomes on attribute level. In addition, we propose simple decision rules to demonstrate the influence of the preference heterogeneity.

## Methods

We used a descriptive survey study design. For reporting, we used the Checklist for Reporting of Survey Studies (CROSS; see Appendix A).^27^ Ethical approval was obtained from the ethics committee domain Humanities and Social Sciences at the University of Twente.

### Attribute Selection

Attributes followed from our overview of eHealth interventions.^18^ For each attribute, first, A.W. and L.B. discussed whether the attribute could be used in the decision rules for the personalization of the intervention advice. If this was the case, it was next discussed whether the attribute was relevant to include in this preference study. Attributes are relevant to include if they are nonredundant, nonoverlapping, and preference independent. Furthermore, the final set of attributes should be complete.^28^ Last, for each included attribute, the levels of the attribute were determined. The levels were determined based on the variation in interventions.^18^ Also, to maintain simplicity, the levels were dichotomized.

The user committee of our PARTNR project consists of various experts in the field of CRF, such as psychologists, oncologists, and patient advocates. They were asked to provide additional input on the attribute selection process as described above. Also, the selected attributes and levels were discussed with these experts. Nine attributes were included for the study (see Table 1). The full list of attributes, including reasons for inclusion or exclusion, is reported in Appendix B.

**Table 1:** Overview of Selected Attributes and Their Levels

Attribute	Binary Levels
Duration	6–12 or 20–26 wk
Sessions per week	Daily or weekly
Time per session	10 min or 1 h
Intervention type	Physical activity or psychosocial
Anonymity	Yes/no
Contact with therapist	Yes/no
Contact with peers	Yes/no
Proven effective^a^	**Yes**/no
Costs^a^	**Yes**/no

a Proven effective and costs are not included in the second part of the survey (within attribute preferences). Instead, it was assumed participants would prefer no costs over costs and a proven effective intervention over a not (yet) proven effective intervention.

### Instrument Design

The survey consisted of three parts. In the first part, we collected background information. In the second part, we elicited within-attribute preferences (i.e., participant preferences regarding the outcomes on attribute level). In the third part, we elicited between-attribute preferences (i.e., the relative importance of attributes; full survey in Appendix C).

In the first part, participants’ age (by birth year), year of breast cancer diagnosis, and the treatment they received for breast cancer were elicited. Using a visual analog scale (VAS; range, 0–10), participants were asked how much they experienced physical, mental, and emotional fatigue, the 3 dimensions of CRF.^29^ Last, interventions already followed for the fatigue were reported, if any.

In the second part, participants were asked to select the attribute level they preferred for each of the attributes (Table 1). Costs and effectiveness were not included as we assumed that participants will always prefer to have no costs and a proven effective intervention.

**Figure 1:**
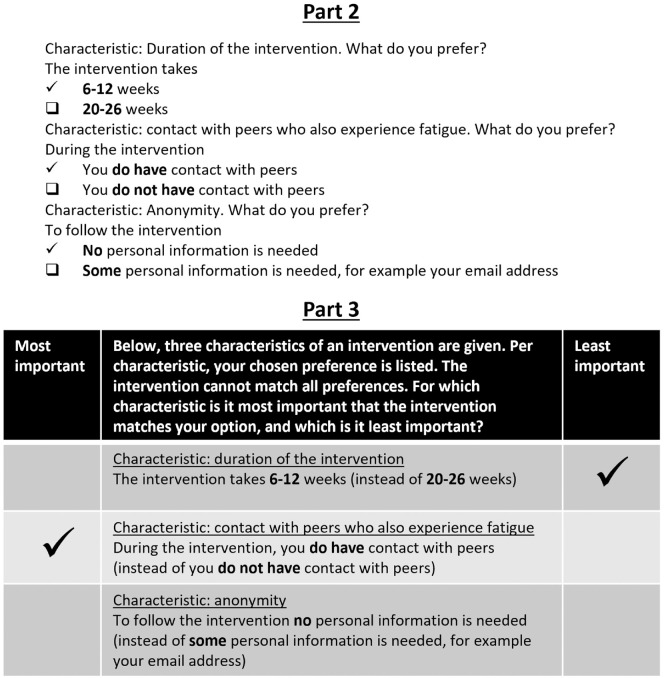
Example of questions in the second and third part of the survey. The actual levels selected in the second part are forwarded to the best-worst scaling in the third part.

In the third part, the relative importance of the attributes was determined using BWS case 1.^30^ BWS allows for analysis of preferences on an individual level.^25^ Participants were asked to compare sets of three attributes and to identify the most and least important from the set. Attributes in each set were varied according to a balanced incomplete block design, so that each attribute and each pair of attributes was presented an equal amount of times.^30^ In our study, participants answered twelve questions, in which each attribute appeared four times and each combination of attributes once. To increase face validity, participants were shown the actual level of the attribute they preferred in part 2. An example is shown in Figure 1.

All text in the survey was written in B1-level Dutch. The wording of the questions on intervention type was checked with an intervention expert, and the full survey was discussed and checked by our two patient advocates of the Dutch Breast Cancer Association (Borstkanker Vereniging Nederland; BVN).

### Study Population

BVN sent out the survey through their social media (Facebook). After the message was posted, participants were given 1 mo to participate in the study (October 20, 2023, to November 24, 2023). Inclusion criteria were being 1) female, 2) diagnosed with breast cancer, and 3) 18 y or older at diagnosis. At the start of the survey, participants provided informed consent and self-reported whether they matched the inclusion criteria. Consent could be withdrawn by quitting the survey. To ensure the privacy of participants, no personal data were collected in the main survey. Once participants finished the survey, in a separate set of questions, participants could leave additional comments and their contact details to receive the results if preferred. The survey was implemented in Qualtrics software, version 10/11 2023.^31^ Qualtrics checked whether participants filled out the survey twice and marked these answers to prevent multiple participation.

### Data Analysis

Data were analyzed in two ways. First, the overall between-attribute and within-attribute preferences were determined. Second, the preference heterogeneity was studied to see the variation in preferences of individuals. The background of the participants was reported using descriptive statistics, based on the first part of the survey.

The between-attribute preferences followed from the BWS ranking questions of the third part of the survey. A counting approach was used: for each individual, scores were calculated based on how often each attribute was selected as *best* (+1) and as *worst* (−1).^32,33^ Each attribute was included in the BWS comparison four times, leading to a score between −4 and 4. The average score with its confidence interval was reported to identify which attributes were overall most and least important.

The within-attribute preferences followed from the second part of the survey. Results of this part were reported by calculating the percentages of participants who chose one outcome over the other. This allowed for identification of the most preferred level per attribute.

### Preference Heterogeneity

To visualize differences in individual between-attribute preferences, the individual BWS scores (between −4 and 4) were transformed to a ranking of the attributes (1–9). In case of ex aequo for two attributes, the next rank was skipped (shared first place: #1, #1, #3 instead of #1, #1, #2). Based on these rankings, a heatmap was created to show how often each attribute/rank combination was chosen by participants. Using this information, the most frequently ranked first attributes can be identified. For within attribute preferences, we looked into what different combinations of outcomes were preferred by participants.

### Decision Rules

With our decision rules, we calculated an individual matching score, linking personal preferences to interventions, based on the attribute levels of the intervention. The simple decision rules consisted of two steps. First, for each attribute, a binary value showed whether the individuals’ within-attribute preference matched the attribute level of the intervention. Second, to incorporate between-attribute preferences, each attribute received a different weighing factor, based on the importance ranking of that attribute, determined from the individual BWS scores. With nine attributes that could be ranked ex aequo, we needed nine linearly scaled weighing factors with an average of 1 (to sum to 100%). Therefore, the highest ranked attribute received a weighing factor of 2, whereas the lowest ranked attribute weighed 0, in steps of 0.25. The weighing factor was multiplied by the binary value from the first step, and the average over all attributes was determined as matching score.

To demonstrate the influence of preference heterogeneity and the need for personalized intervention advice, we calculated participants’ matching score for hypothetical interventions. Attributes of a first hypothetical intervention corresponded to the overall within-attribute preferences of our participant group (see the “Results” section). For additional hypothetical interventions, we changed the binary level of one of the nine attributes (Table 1) to the other option, nine times, one at the time. Then, for each individual, it was determined which of these 10 hypothetical interventions had the highest matching score based on the decision rules.

All analyses were performed in Python version 3.11.9. No comparison could be made between the participants who did and did not finish the survey. Not finishing the survey meant that participants also withdrew their informed consent. We recorded at what part of the survey participants dropped out.

## Results

### Study Population

**Figure 2:**
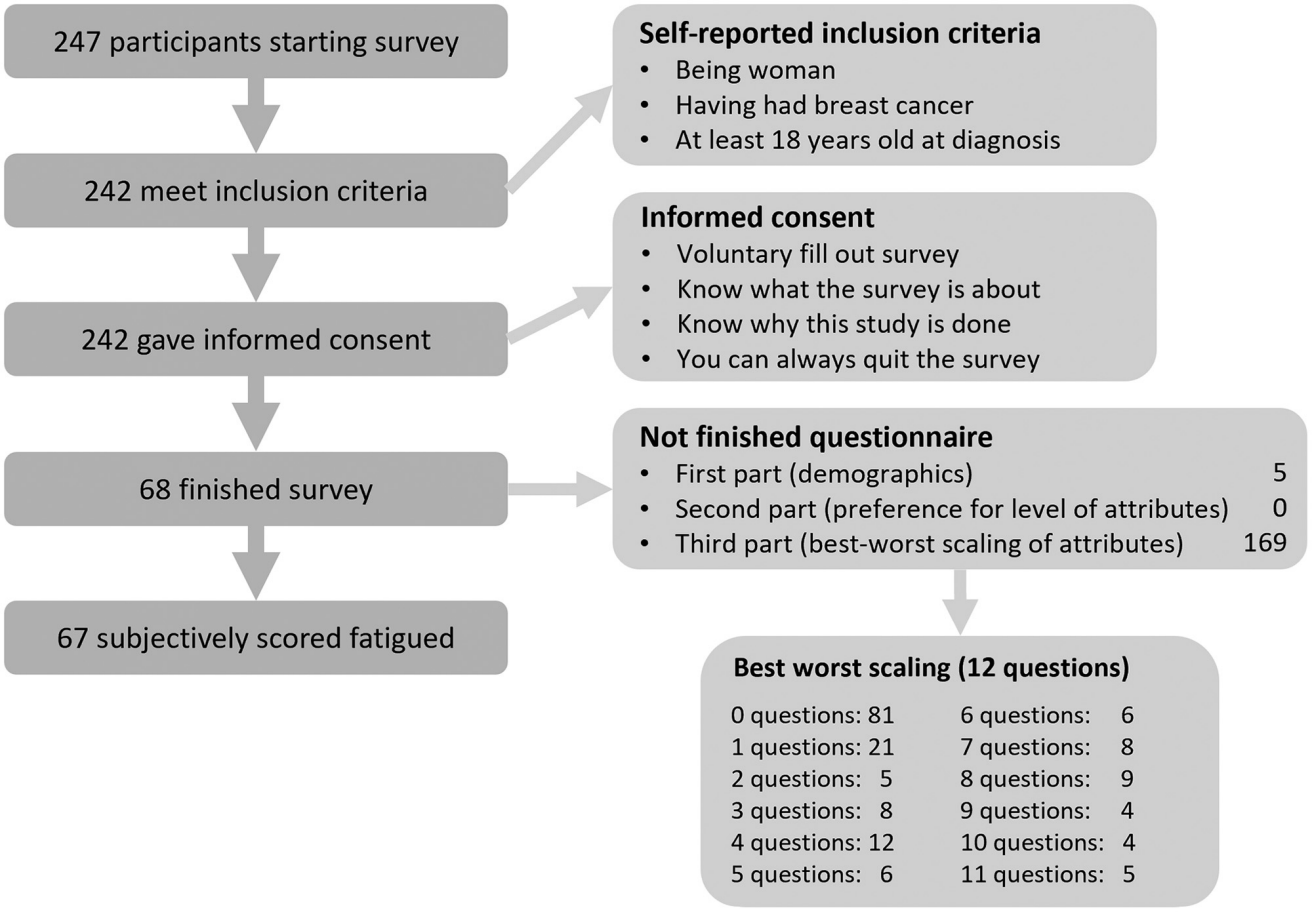
Flow of participants in the survey.

In total, 247 participants started the survey. Sixty-seven (27%) finished the survey and were included in the analysis. It took them a median of 9.4 min to fill out the survey (Appendix D shows the boxplot of duration). Figure 2 shows the flow of participants and in what part of the survey participants dropped out. One participant was excluded after finishing the survey, because she scored no fatigue on any of the dimensions.

On average, participants were 52 y old and were 4.5 y after their breast cancer diagnosis. All participants had surgery, and 99% (*n* = 66) received at least 1 additional treatment, of which radiotherapy (*n* = 59, 88%) was reported most. On average, participants experienced moderate to severe mental (7.2/10), emotional (6.6/10), and physical fatigue (6.5/10). Less than half of the participants (*n* = 29, 43%) already followed an intervention for fatigue; some participants mentioned having tried several interventions (Table 2).

**Table 2:** Background of Participants

Characteristic	$\bar{x}$ (*s*) or No. (%)
Age, y	
At filling in survey	51.6 (8.0)
At diagnosis breast cancer	47.1 (9.0)
Time since diagnosis	4.5 (4.0)
Treatment	
Surgery	67 (100%)
Chemotherapy	44 (66%)
Radiotherapy	59 (88%)
Hormonal therapy	46 (69%)
Immunotherapy	7 (10%)
Targeted therapy	1 (1%)
Self reported fatigue (visual analog scale 0–10)	
Physical fatigue	6.5 (2.3)
Mental fatigue	7.2 (2.1)
Emotional fatigue	6.6 (2.4)
Treatment for fatigue	29 (43%)
Psychological	8 (28% of 29 who followed an intervention)
Physiotherapy	9 (31%)
(Oncologic) rehabilitation program	10 (34%)
Acupuncture	4 (14%)
Occupational therapy	6 (21%)
Other	9 (31%)

### Between-Attribute and within-Attribute Preferences

**Figure 3:**
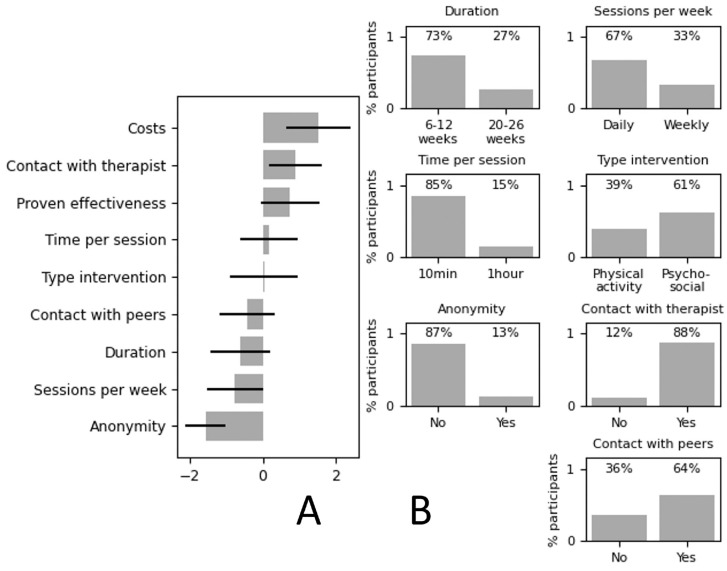
Overall preferences of the participants. (A) Between-attribute preferences showing the relative importance of the attributes with the corresponding 95% confidence interval. (B) Within-attribute preferences showing preferences for the levels of the attributes. Variance is shown based on percentages of participants preferring one level over the other.

Overall, participants ranked *costs* as the most important attribute and *anonymity* as the least important attribute (Figure 3A). On average, participants preferred shorter treatment duration, daily sessions, shorter time per session, and a psychosocial instead of a physical intervention. Also, they had no need to stay anonymous and preferred contact with a therapist and peers (see Figure 3B).

### Preference Heterogeneity

**Figure 4:**
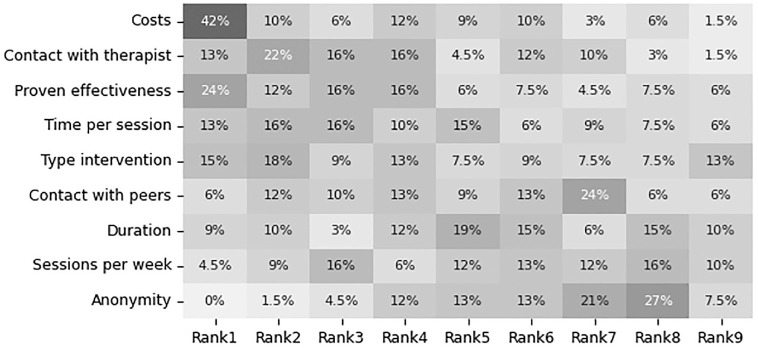
Heatmap of all participants and how often they ranked an attribute at a certain position. Costs and proven effectiveness were ranked first most often. Anonymity was never ranked first by any of the participants.

The 3 attributes that were ranked most frequently as most important attribute were *costs* (*n* = 28, 42%), *proven effectiveness* (*n* = 16, 24%), and *type of intervention* (*n* = 10, 15%, Figure 4). For the other rankings and attributes, Figure 4 also shows how participants ranked the various attributes. In Appendix D, we show how each individual participant ranked the attributes. This shows the extent of the heterogeneity, as none of the participants ranked all attributes in the same order.

For within-attribute preferences, preference heterogeneity was smallest for shorter time per session, no need to stay anonymous, and contact with the therapist. The most frequent preference pattern (combination of within-attribute preferences) overlapped with the overall within-attribute preferences and was preferred by 13 (19%) participants. Some participants (31%) deviated from the most common preference pattern on only one attribute: 8 participants (12%) preferred to have no contact with peers, whereas 5 (7%) participants preferred a physical activity intervention instead of a psychosocial intervention. Most participants (49%) had different preferences for two or more attribute levels: 3 (4%) participants preferred weekly sessions and longer time per session, whereas 2 (3%) participants preferred weekly sessions, a physical activity intervention, and wanted to stay anonymous without contact with a therapist nor peers.

### Decision Rules

**Figure 5:**
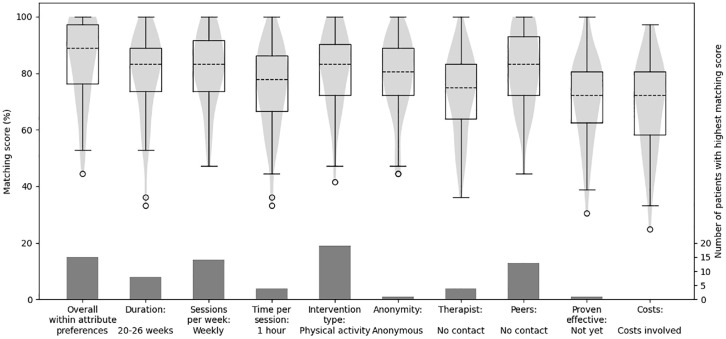
Violin plot overlapped by boxplot showing the variation in matching scores for all 67 participants. The first violin plot corresponds to a hypothetical intervention that has a perfect match with the overall within-attribute preferences as reported in this article. The other 9 hypothetical interventions vary from this first intervention by changing 1 of the attributes to the other binary level. The bars show the amount of participants who had the highest matching score for that specific intervention.

Based on our simple decision rules, the matching scores with the first hypothetical intervention ranged from 44% to 100% (Figure 5, left-most violin plot). In Appendix D, we show for one participant how the matching score was calculated. The attributes of this first hypothetical intervention corresponded to the overall within-attribute preferences of shorter treatment duration, daily sessions, shorter time per session, and a psychosocial instead of a physical intervention. Also, this intervention required personal data (no anonymity) and involved contact with a therapist and peers. Last, the intervention was proven effective, and no costs were involved.

Next, we changed the binary level of each of the attributes of this first hypothetical intervention, one at the time, to create another nine additional hypothetical interventions. For all nine hypothetical interventions, except when the level no costs changed to costs involved, at least one participant had a matching score of 100% (Figure 5, other violin plots). For 15 participants (22%), the highest matching score was with the first hypothetical intervention. For the others, the highest score was with one of the other nine hypothetical interventions. For example, if the *type of intervention* changed to physical activity, 19 participants (28%) had their highest matching score with this adjusted intervention (Figure 5). It could be that one individual had two interventions with the highest score, as the least important attribute had a weight of zero. This means that whether the within-attribute preference overlaps with the intervention (as determined in step 1) is ignored. For example, if a participant preferred weekly sessions over daily sessions but ranked sessions per week at rank 9, then an intervention with daily sessions and an intervention with weekly sessions would have the same matching score.

## Discussion

To work toward personalized intervention advice for CRF after breast cancer, the aim of this study was to explore preference heterogeneity regarding 1) the relative importance of intervention attributes and 2) outcomes on attribute level. In addition, we proposed simple decision rules to demonstrate the influence of the preference heterogeneity.

We elicited preferences related to attributes of eHealth interventions for CRF from 67 breast cancer survivors using BWS. Participants ranked costs as the most important and anonymity as the least important attribute. Regarding preference heterogeneity, participants ranked *costs, proven effectiveness*, and *type of intervention* as most important most frequently. For outcomes on attribute level, participants preferred shorter treatment duration, daily sessions, shorter time per session, and a psychosocial intervention. They had no need to stay anonymous and preferred contact with a therapist and peers. Analysis of preference heterogeneity showed that individual preferences could differ on one or more attributes.

Using our simple decision rules, we showed the influence of preference heterogeneity on intervention advice based on a matching score. For 22% of the participants, an intervention with the attributes matching the overall within-attribute preferences had the highest matching score. For the others (*n* = 52, 78%), a higher matching score was reached when the level of 1 of the attributes was changed.

### Implications

The large extent of heterogeneity in preferences for attributes of CRF interventions shows the need for personalization. Personalization and tailoring are already included in interventions. Van der Leeden et al.^34^ developed a framework to adjust exercise interventions during chemotherapy to experienced side effects or comorbidities. The *Cancer Aftercare Guide*^35^ uses a screening questionnaire to determine what specific modules on long-term effects after cancer are relevant for individual patients. *StressProffen* incorporated preferences by allowing users to mark their favorite exercises, and their app can be used on both a tablet and smartphone to accommodate for age-related differences in preferences.^36^ Harnas et al.^37^ aimed to personalize a cognitive behavior therapy based on patient preferences for four different aspects: the symptom of focus, treatment delivery, treatment modules, and treatment duration.

Preference heterogeneity exists on more than one attribute. As there is also variation in eHealth interventions for CRF after breast cancer,^18^ this inquires a new approach in which patients are matched to an already existing intervention. With our decision rules, we demonstrated that, due to the extent of preference heterogeneity, developing a new intervention does not lead to the optimal intervention for all targeted individuals. For more than half of the participants, changing one of the attributes to the other level led to a higher matching score. Which intervention was the best match depended on the preferences of an individual. Therefore, instead of developing a new intervention, the focus should be on supporting patients by helping them find an existing intervention that fits them best.

In clinical practice, decision rules and matching scores can be used in a shared decision-making context. The matching scores and preferences can be a starting point for a conversation between the patient and health care professional on CRF after breast cancer. Preferences might change over time, as patients gain new knowledge or the experience of an intervention teaches them more about their preferences.^38^ Sava et al.^38^ developed a framework to support decision making allowing for changing preferences. This framework is also relevant to consider with our decision rules.

### Strengths and Limitations

Often, studies evaluate heterogeneity on a group level. In the analysis of preference heterogeneity, we focused on the individual patient. By showing how many individuals ranked a certain attribute at a certain position, we considered individual differences. Also, by calculating and comparing the example interventions on individual matching scores, the focus is on individuals. This is a strength of this study.

Only 27% of the people who started the survey also finished. Most participants quit in the last part of the survey, which contained the BWS (Figure 2). We took various measures to simplify the survey; nevertheless, it is unclear for what reason participants did not finish the survey. We selected the attributes and levels in consultation with experts from the fields of CRF and patient preferences; however, we did not perform a qualitative study to include patients in this process. Possibly, this could have improved the survey and, with that, the completion rate. Methods other than BWS might have improved our results in terms of completion rate and hence sample size. However, we were specifically interested in individual preferences, which reduced the methods to choose from. AHP could be an alternative; however, with 9 attributes, participants have to weigh 36 combinations of attributes whereas we had 19 questions (seven in part 2, twelve in part 3).

Our relatively small, nongeneralizable sample size limits the generalizability of the results. The participants were relatively young, with an average age of 47 y at diagnosis, where most women in the Netherlands are older than 50 y at diagnosis.^39^ Recruitment was carried out via the Facebook page of BVN, the breast cancer association of the Netherlands. Other studies that used Facebook to recruit participants also had a relatively small sample size. These studies recruited over a longer period and used more posts and Facebook advertorial services.^40,41^

In the survey, we did not collect any personal information of the participants, so we had no opportunity to verify whether participants matched the inclusion criteria, other than their self-reported information. However, we checked the time participants took to finish the questionnaire (median 9.4 min, boxplot in Appendix D), and there were no outliers in terms of a too fast completion time. Also, participants reported their year of diagnosis, treatment received, and experience with CRF as additional checks for the inclusion criteria. Finally, 41 participants (61%) left their contact details to receive results and/or information on future studies. Altogether, even though we could not check that participants matched the inclusion criteria, we can at least assume that most participants were honest in their participation.

Still, we were able to show preference heterogeneity and the variation in matching scores using the simple decision rules. With a larger, more generalizable sample size, the heterogeneity in preferences would still be present. This is also why no sample size was calculated.

Our study could suffer from selection bias, as we reached only those who follow BVN on Facebook.^40^ In addition, nonresponse bias might have influenced our results, as many participants did not finish the survey and, with that, withdrew their consent. We could not analyze their responses and compare whether these were different from completed responses.

### Future Research

There are several aspects we want to consider in the further development of our decision rules before they can be implemented in clinical practice. The current version is simple and based on a linear scaled weighing factor between 0 and 2.

In the example interventions, the intervention attributes matched the binary levels of the preference attributes perfectly. When using existing interventions to calculate matching scores, this is not always the case. For example, an intervention can last 16 wk (instead of the categories 6–12 wk or 20–26 wk), have 3 sessions per week (instead of daily or weekly), or have sessions of 30 min (instead of 10 min or 1 h). Next to patient preferences, it is also important to acknowledge individuals’ needs. Using a holistic approach,^42^ these can be mapped and included in the decision rules as well. Further, the expected effectiveness of an intervention on the individual level is important. It could be that an individual prefers a physical activity intervention but a psychological intervention would be more effective and fit their needs better.

Already during the development process of the decision rules, patients and health care professionals need to be asked for their input. Once all of the above-mentioned aspects are considered, the decision rules also need to be validated and certified. Only then can the decision rules be implemented in clinical practice.

## Conclusion

In this study on breast cancer patients’ preferences for attributes of CRF interventions, we looked into preference heterogeneity and the extent of this heterogeneity. We found heterogeneity in between-attribute and within-attribute preferences. Participants ranked different attributes as most important. This happened most often for *costs, proven effectiveness*, and *type of intervention*. Overall, participants chose a shorter intervention, daily sessions, shorter time per session, a psychosocial intervention, no need for anonymity during the intervention, and contact with a therapist and peers. Thirteen (19%) participants had this preference pattern of within-attribute preferences, and the other participants varied on 1 or more attribute outcomes.

We also demonstrated the influence of preference heterogeneity on matching scores for several interventions by using simple decision rules. For 10 example interventions, we calculated matching scores for all participants to show that an intervention with attributes that correspond to the overall within-attribute preferences is not the best matching intervention for all participants.

Our results underline the need for personalization of care; it is imperative to tailor intervention advice to individual patient preferences. This will improve the chance of higher adherence to and outcomes of CRF interventions. These improved outcomes will reduce the burden of CRF after breast cancer and subsequently improve the quality of life after cancer.

## Supplemental Material

sj-pdf-1-mpp-10.1177_23814683241309676 – Supplemental material for Working toward Personalized Intervention Advice: A Survey Study on Preference Heterogeneity in Patients with Breast Cancer–Related Fatigue Supplemental material, sj-pdf-1-mpp-10.1177_23814683241309676 for Working toward Personalized Intervention Advice: A Survey Study on Preference Heterogeneity in Patients with Breast Cancer–Related Fatigue by Lian Beenhakker, Kim A. E. Wijlens, Christina Bode, Miriam M. R. Vollenbroek-Hutten, Sabine Siesling, Janine A. van Til and Annemieke Witteveen in MDM Policy & Practice
