# Supplementary material for: Working toward Personalized Intervention Advice: A Survey Study on Preference Heterogeneity in Patients with Breast Cancer–Related Fatigue
Source: MDM Policy Pract. 2025 Jan 13;10(1):23814683241309676. doi: 10.1177/23814683241309676 (PMC11726506; doi:10.1177/23814683241309676)
Supplement: sj-pdf-1-mpp-10.1177_23814683241309676 – Supplemental material for Working toward Personalized Intervention Advice: A Survey Study on Preference Heterogeneity in Patients with Breast Cancer–Related Fatigue [file sj-pdf-1-mpp-10.1177_23814683241309676.pdf]

Appendices

Appendix A – CROSS checklist

Checklist for Reporting Of Survey Studies (CROSS)

Section/topic	Item	Item description	Reported on page #
**Title and abstract**			
Title and abstract	1a	State the word "survey" along with a commonly used term in title or abstract to introduce the study's design.	Title
	1b	Provide an informative summary in the abstract, covering background, objectives, methods, findings/results, interpretation/discussion, and conclusions.	Abstract
**Introduction**			
Background	2	Provide a background about the rationale of study, what has been previously done, and why this survey is needed.	Introduction
Purpose/aim	3	Identify specific purposes, aims, goals, or objectives of the study.	Introduction
**Methods**			
Study design	4	Specify the study design in the methods section with a commonly used term (e.g., cross-sectional or longitudinal).	Methods
Data collection methods	5a	Describe the questionnaire (e.g., number of sections, number of questions, number and names of instruments used).	Instrument design
	5b	Describe all questionnaire instruments that were used in the survey to measure particular concepts. Report target population, reported validity and reliability information, scoring/classification procedure, and reference links (if any).	Instrument design
	5c	Provide information on pretesting of the questionnaire, if performed (in the article or in an online supplement). Report the method of pretesting, number of times questionnaire was pre-tested, number and demographics of participants used for pretesting, and the level of similarity of demographics between pre-testing participants and sample population.	Appendix B
	5d	Questionnaire if possible, should be fully provided (in the article, or as appendices or as an online supplement).	Appendix C
Sample characteristics	6a	Describe the study population (i.e., background, locations, eligibility criteria for participant inclusion in survey, exclusion criteria).	Methods – Study population
	6b	Describe the sampling techniques used (e.g., single stage or multistage sampling, simple random sampling, stratified sampling, cluster sampling, convenience sampling). Specify the locations of sample participants whenever clustered sampling was applied.	Methods – Study population

	6c	Provide information on sample size, along with details of sample size calculation.	Limitations
	6d	Describe how representative the sample is of the study population (or target population if possible), particularly for population-based surveys.	Limitations
Survey administration	7a	Provide information on modes of questionnaire administration, including the type and number of contacts, the location where the survey was conducted (e.g., outpatient room or by use of online tools, such as SurveyMonkey).	Methods – Study population
	7b	Provide information of survey's time frame, such as periods of recruitment, exposure, and follow-up days.	Methods – Study population
	7c	Provide information on the entry process:<br>–>For non-web-based surveys, provide approaches to minimize human error in data entry.<br>–>For web-based surveys, provide approaches to prevent "multiple participation" of participants.	Methods – Study population
Study preparation	8	Describe any preparation process before conducting the survey (e.g., interviewers' training process, advertising the survey).	N.A.
Ethical considerations	9a	Provide information on ethical approval for the survey if obtained, including informed consent, institutional review board [IRB] approval, Helsinki declaration, and good clinical practice [GCP] declaration (as appropriate).	Methods
	9b	Provide information about survey anonymity and confidentiality and describe what mechanisms were used to protect unauthorized access.	Methods – Study population
Statistical analysis	10a	Describe statistical methods and analytical approach. Report the statistical software that was used for data analysis.	Data analysis
	10b	Report any modification of variables used in the analysis, along with reference (if available).	Data analysis
	10c	Report details about how missing data was handled. Include rate of missing items, missing data mechanism (i.e., missing completely at random [MCAR], missing at random [MAR] or missing not at random [MNAR]) and methods used to deal with missing data (e.g., multiple imputation).	N.A.
	10d	State how non-response error was addressed.	Data analysis
	10e	For longitudinal surveys, state how loss to follow-up was addressed.	N.A.
	10f	Indicate whether any methods such as weighting of items or propensity scores have been used to adjust for non-representativeness of the sample.	N.A.
	10g	Describe any sensitivity analysis conducted.	N.A.
**Results**			

Respondent characteristics	11a	Report numbers of individuals at each stage of the study. Consider using a flow diagram, if possible.	Figure 2
	11b	Provide reasons for non-participation at each stage, if possible.	N.A.
	11c	Report response rate, present the definition of response rate or the formula used to calculate response rate.	Results – Study population
	11d	Provide information to define how unique visitors are determined. Report number of unique visitors along with relevant proportions (e.g., view proportion, participation proportion, completion proportion).	Figure 2
Descriptive results	12	Provide characteristics of study participants, as well as information on potential confounders and assessed outcomes.	Table 2
Main findings	13a	Give unadjusted estimates and, if applicable, confounder-adjusted estimates along with 95% confidence intervals and p-values.	Figure 3
	13b	For multivariable analysis, provide information on the model building process, model fit statistics, and model assumptions (as appropriate).	N.A.
	13c	Provide details about any sensitivity analysis performed. If there are considerable amount of missing data, report sensitivity analyses comparing the results of complete cases with that of the imputed dataset (if possible).	N.A.
**Discussion**			
Limitations	14	Discuss the limitations of the study, considering sources of potential biases and imprecisions, such as non-representativeness of sample, study design, important uncontrolled confounders.	Limitations
Interpretations	15	Give a cautious overall interpretation of results, based on potential biases and imprecisions and suggest areas for future research.	Discussion
Generalizability	16	Discuss the external validity of the results.	Limitations
**Other sections**			
Role of funding source	17	State whether any funding organization has had any roles in the survey's design, implementation, and analysis.	Acknowledge ments
Conflict of interest	18	Declare any potential conflict of interest.	Conflict of Interest
Acknowledge-ments	19	Provide names of organizations/persons that are acknowledged along with their contribution to the research.	Acknowledge ments

Appendix B – Attribute selection

Possible attributes	Useful in intervention advice	Useful to include in questionnaire	Remarks
**Anonymous**	Yes	Yes	Relevant, included as yes/no
**Category intervention**<br>**Type of intervention**	Yes	Yes	Relevant, included as physical activity or psychosocial
**Costs**	Yes	Yes	Relevant, included as yes/no
**Duration intervention**	Yes	Yes	Relevant, included as 6-12 or 20-26 weeks
**Contact with therapist**	Yes	Yes	Relevant, included as yes/no
**Contact with peers**	Yes	Yes	Relevant, included as yes/no
**Prescribed intensity**	Yes	Yes	Relevant, related to time per session and sessions per week, included as 10 min or 1 hour and daily or weekly
**Primary results / effectiveness**	Yes	Yes	Relevant, included as yes/no
**% BC patients**	Yes	No	Related to generalizability of the effect, use as weighing factor
**(A)synchronous**	Yes	No	Relevant for advice, related to contact with healthcare professional and content delivery, not useful in questionnaire
**Adherence characteristics**	Yes	No	Related to effectiveness of intervention, use as weighing factor
**Age range**	Yes	No	Related to inclusion criteria, use as weighing factor
**All in/exclusion criteria**	Yes	No	Include whether patient matches original criteria of intervention, use as weighing factor
**Content delivery**	Yes	No	Interventions can have combinations of content delivery, relevant for advice, not for questionnaire
**Demographics related to treatment**	Yes	No	Related to inclusion criteria, use as weighing factor
**Dropout reasons**<br>**(not finishing intervention)**	Yes	No	Related to effectiveness of intervention, use as weighing factor
**Fatigue included as primary or secondary outcome measure**	Yes	No	Related to whether effect for fatigue was goal of intervention, use as weighing factor
**Intervention relative to treatment**	Yes	No	Related to inclusion criteria, use as weighing factor
**Language**<br>**Country of intervention**	Yes	No	Relevant, pilot study showed that patients prefer their native language. Useful to show users what language the intervention has, not for questionnaire
**Patient characteristics (successful)**<br>**Other relations with characteristics**	Yes	No	Related to effectiveness of intervention, use as weighing factor
**Reasons for dropout**	Yes	No	Relevant to explain possible reasons why previous patients did not finish the intervention

**Results at follow-up**	Yes	No	Related to effectiveness of intervention over time, not known for all interventions, use as possible weighing factor
**Total number of participants**	Yes	No	Related to generalizability of the effect, use as weighing factor
**Type of study**	Yes	No	Related to effectiveness of intervention, use as weighing factor
**>5 years after cancer treatment**	No	No	Related to inclusion criteria of intervention relative to treatment, not relevant on its own
**0-5 years after cancer treatment**	No	No	Related to inclusion criteria of intervention relative to treatment, not relevant on its own
**Author/Year**	No	No	Not related to following an intervention.
**Contact with research team**	No	No	Not realistic in non-research setting
**Description category**	No	No	Detailed description of the category of the intervention, not related to intervention advice
**Description intervention**	No	No	Relevant to explain the intervention to the patient
**Duration follow-up**	No	No	Related to effectiveness of intervention over time, not known for all interventions, not relevant on its own
**During cancer treatment**	No	No	Related to inclusion criteria of intervention relative to treatment, not relevant on its own
**Experiences with intervention**	No	No	Relevant to explain how previous patients experienced the intervention, not for personalization of intervention advice
**How to find the intervention?**	No	No	Relevant to explain the intervention to the patient, not for personalization of intervention advice
**Human contact**	No	No	Divided into contact with research team and healthcare professional, not relevant as combined attribute
**Introduction to intervention**	No	No	Relevant to explain the intervention to the patient, not for personalization of intervention advice
**Name intervention**	No	No	Relevant to explain the intervention to the user, not for personalization of intervention advice
**Proof for choice of type of intervention**	No	No	Detailed description of the category of the intervention and the references that were used to support this choice of category
**Questionnaire fatigue**	No	No	All methods are validated questionnaires
**Recruitment of patients**	No	No	Not related to following an intervention.
**Usage / adherence**	No	No	Relevant to explain how well previous patients used/adhered to the intervention, not for personalization of intervention advice
**Year of publication**	No	No	Not related to following an intervention.

Pilot study

Prior to our study described in this manuscript, a pilot study was performed. In this pilot study, six breast cancer patients recruited from our own network were asked for their preferences regarding several attributes and their levels. Their age ranged 48-72. Two participants reported mental fatigue, two participants reported physical fatigue and two participants reported to experience no CRF. Eight preference-sensitive attributes were selected from the attributes in this appendix.

The attributes and outcomes were as follows: duration (5 levels, 6-26 weeks), intensity (5 levels, daily to monthly), type of intervention (2 levels, physical activity or psychosocial), language of intervention (4 levels, Dutch, English, German or French), contact (4 levels, no contact, with therapist, with research team, or with peers), costs involved (2 levels, yes, no), anonymity (2 levels, yes, no), proven effectiveness (2 levels, yes, not yet). Participants were asked to rank the levels per attribute and weigh the importance of the attributes to each other using the Analytic Hierarchy Process (AHP) [A1, A2]. In this setting, participants were asked to weigh the levels and attributes in pairs, leading to over fifty comparisons.

The results of this pilot study already confirmed our hypothesis that preferences vary between participants. However, the consistency analysis of the AHP methodology showed that only one of the six patients was consistent in comparing all pairs in the survey. Therefore, for the study in this manuscript, we reconsidered the attribute selection and survey setup.

In comparison to the outcomes in the pilot study, we removed language of the intervention, as patients preferred their native language. Intensity was divided into sessions per week and time per session. Contact was divided into contact with therapist and contact with peers.

[A1] T. L. Saaty, 'Decision making with the analytic hierarchy process', *International Journal of Services Sciences*, vol. 1, no. 1, p. 83, 2008, doi: 10.1504/IJSSCI.2008.017590.

[A2] L. Abdullah, I. Taib, and R. Salleh, 'Public perceptions of cancer risk using analytic hierarchy process', *Journal of Applied Sciences*, vol. 9, no. 12, pp. 2319–2324, 2009, doi: 10.3923/jas.2009.2319.2324.

Appendix C – Full questionnaire (translated to English, originally in Dutch)

Part 1

1) What is your year of birth?
2) What year did you receive the diagnosis of breast cancer?
3) What treatment(s) did you receive for breast cancer?
   - Surgery
   - Chemotherapy
   - Radiotherapy
   - Hormonal therapy
   - Other: [open question]
4) What was the last moment you received these treatments?
   - Surgery
   - Chemotherapy
   - Radiotherapy
   - Hormonal therapy
   - [Insert answer of open question]
5) On a scale from 0-10, how much do you experience fatigue after the diagnosis and treatment of breast cancer?
   - Physical fatigue
   - Mental fatigue
   - Emotional fatigue
6) Did you follow an intervention for fatigue? If yes, what type of intervention?

Part 2

Below are several characteristics of an intervention for fatigue. Per characteristic, there are two options. Please fill out what option you prefer.

1) Characteristic: Duration of the intervention. What do you prefer?
   The intervention takes
   - ☐ **6-12** weeks
   - ☐ **20-26** weeks
2) Characteristic: Number of sessions per week. What do you prefer?
   It is expected that you train
   - ☐ **Every day**
   - ☐ **Once a week**
3) Characteristic: duration of one session. What do you prefer?
   One session takes
   - ☐ Around **10 minutes**
   - ☐ Around **1 hour**
4) Characteristic: goal of the intervention. What do you prefer?
   The intervention focuses on
   - ☐ Feeling **physically more fit**
   - ☐ How to better **deal with fatigue**
5) Characteristic: Anonymity. What do you prefer?
   To follow the intervention
   - ☐ **No** personal information is needed
   - ☐ **Some** personal information is needed, for example your email address
6) Characteristic: Contact with a healthcare professional (for example, calling or emailing).
   What do you prefer?

You can also always visit your general practitioner if you experience complaints.
During the intervention,
- ☐ You **do have** contact with a healthcare professional
- ☐ You **do not have** contact with a healthcare professional

7) Characteristic: contact with peers who also experience fatigue. What do you prefer?
During the intervention
- ☐ You **do have** contact with peers
- ☐ You **do not have** contact with peers

Part 3

Below, three characteristics of an intervention are given. Per characteristic, your chosen preference is listed. The intervention cannot match all preferences. For which characteristic is it most important that the intervention matches your option and which is it least important?

Depending on the chosen option in part 2, below, either of the two texts in shown in part 3.

1. <u>Characteristic: Duration of the intervention</u>
   - The intervention takes **6-12** weeks (instead of **20-26** weeks)
   - The intervention takes **20-26** weeks (instead of **6-12** weeks)

2. <u>Characteristic: Number of sessions per week</u>
   - It is expected that you train **every day** (instead of **once a week**)
   - It is expected that you train **once a week** (instead of **every day**)

3. <u>Characteristic: duration of one session</u>
   - One session takes around **10 minutes** (instead of around **1 hour**)
   - One session takes around **1 hour** (instead of around **10 minutes**)

4. <u>Characteristic: goal of the intervention</u>
   - The intervention focusses on feeling **physically more fit** (instead of how to better **deal with fatigue**)
   - The intervention focusses on how to better **deal with fatigue** (instead of feeling **physically more fit**)

5. <u>Characteristic: Anonymity</u>
   - To follow the intervention **no** personal information is needed (instead of **some** personal information is needed, for example your email address)
   - To follow the intervention **some** personal information is needed, for example your email address (instead of **no** personal information is needed)

6. <u>Characteristic: Contact with a healthcare professional</u>
   - During the intervention, you **do have** contact with a healthcare professional (instead of you **do not have** contact with a healthcare professional)
   - During the intervention, you **do not have** contact with a healthcare professional (instead of you **do have** contact with a healthcare professional)

7. <u>Characteristic: contact with peers who also experience fatigue.</u>
   - During the intervention, you **do have** contact with peers (instead of you **do not have** contact with peers)
   - During the intervention, you **do not have** contact with peers (instead of you **do have** contact with peers)

8. Characteristic: proven effectiveness of the intervention
    - Research **did show** that the intervention works (instead of **did not yet show** the intervention works)

9. Characteristic: costs involved for the intervention
    - There are **no costs involved** when following the intervention (instead of there are **costs involved** when following the intervention)

The table below shows how the attributes are being combined. There are twelve questions in which three attributes are being compared. The order in which participants see the twelve combinations is randomized as well as the order of the three attributes in the question.

1, 2, 3	1, 4, 5
1, 6, 7	1, 8, 9
2, 4, 7	2, 5, 8
2, 6, 9	3, 4, 9
3, 5, 6	3, 7, 8
4, 6, 8	5, 7, 9

Appendix D – Additional information

Additional figures

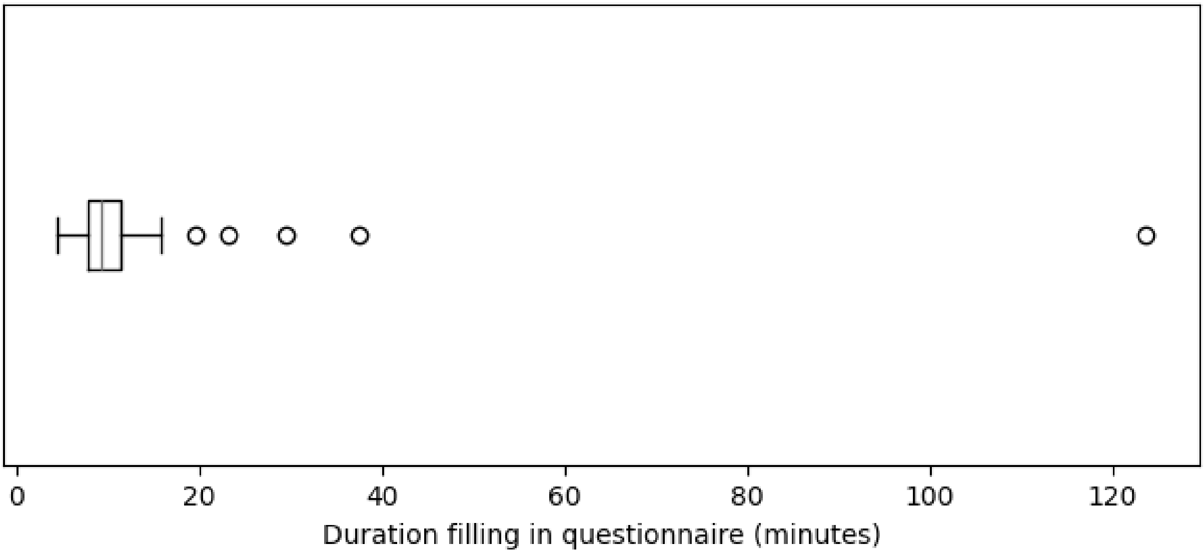

Figure 1 – Boxplot of the time it took participants to fill out the questionnaire. The minimum value is 4.5 minutes and there are four outliers with longer duration.

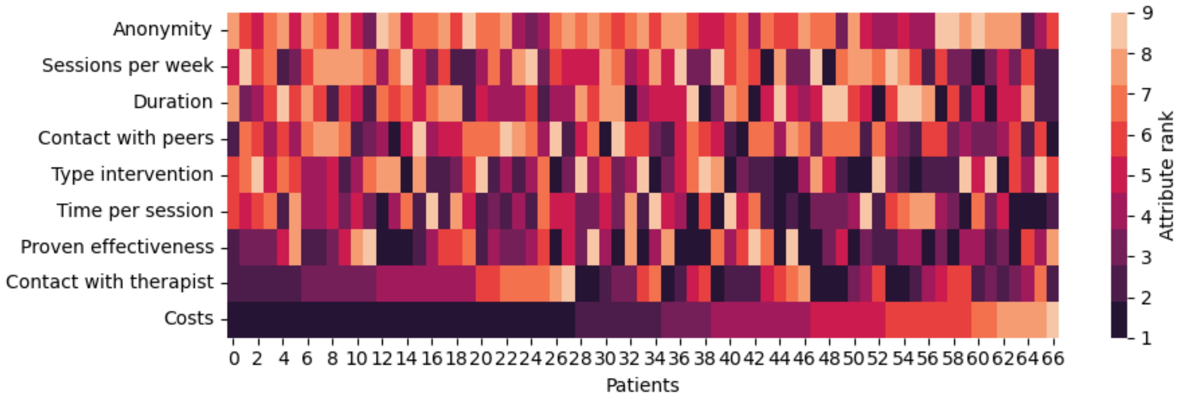

Figure 2 – Ranking of attributes per individual participant. On the x-axis, all participants are presented, the y-axis shows the attributes and the color shows the ranking: darker means higher ranked.

Calculation matching score

As described in the methods section, the decision rules consisted of two steps:

- First, for each attribute, a binary value showed whether the individuals' within attribute preference matched the attribute level of the intervention.
- Second, to incorporate between attribute preferences, each attribute received a different weighing factor, based on the importance ranking of that attribute, determined from the individual BWS scores. With nine attributes that could be ranked ex aequo, we needed nine linearly scaled weighing factors with an average of 1 (to sum to 100%). Therefore, the highest

ranked attribute received a weighing factor of '2', whereas the lowest ranked attribute weighs '0', in steps of '0.25' (see table below).

**Rank**	1	2	3	4	5	6	7	8	9
**Weighing factor**	2	1.75	1.5	1.25	1	0.75	0.5	0.25	0

Then, the weighing factor was multiplied by the binary value from the first step, and the average over all attributes was determined as matching score.

The overview on the below shows this process for one individual.

**Intervention attributes**		**Preferences**		**Step 1**	**Step 2**	**Combine**
Attributes	*Binary level*	*Within attributes*	*Between attributes*			
**Duration**	6-12 weeks	6-12 weeks	6	1	0.75	0.75
**Sessions per week**	Daily	Daily	9	1	0	0
**Time per session**	10 min	10 min	2	1	1.75	1.75
**Type of intervention**	Psychosocial	Physical activity	4	0	1.25	0
**Anonymity**	No	No	3	1	1.5	1.5
**Contact with therapist**	Yes	Yes	7	1	0.5	0.5
**Contact with peers**	Yes	No	7	0	0.5	0
**Proven effective**	Yes	Yes*	4	1	1.25	1.25
**Costs**	No	No*	1	1	2	2
						**SUM: 7.75**<br>**Match: 86%**

* Proven effective and costs were not included in the second part of the questionnaire (within attribute preferences). Instead, it was assumed participants would prefer no costs over costs and a proven effective intervention over a not (yet) proven effective intervention.
